# Effects of dietary protein restriction followed by realimentation on growth performance and liver transcriptome alterations of lamb

**DOI:** 10.1038/s41598-018-33407-w

**Published:** 2018-10-12

**Authors:** K. Cui, B. Wang, T. Ma, B. W. Si, N. F. Zhang, Y. Tu, Q. Y. Diao

**Affiliations:** Feed Research Institute, Chinese Academy of Agricultural Sciences, National Engineering Research Center of Biological Feed, Beijing, China

## Abstract

The present study aimed to investigate the compensatory effect of early protein restriction followed by a realimentation on growth performance of lamb and to explore the transcriptomic changes in liver. Thirty-two lambs with an initial birth weight of 2.3 ± 0.20 kg that were weaned on day 15 were randomly divided into two groups. The lambs were fed a basal diet with normal protein level (NPL, protein level in the milk replacer and starter, 25 and 21%, respectively) or low protein level (LPL, protein level in the milk replacer and starter, 19 and 15%, respectively) from 15 to 60 d, after which all lambs consumed the same diet with a normal protein level from 61 to 90 d. Protein restriction led to a significant decrease in average daily gain (ADG), body weight and liver weight (*P* < 0.05). Transcriptome analysis showed that 302 or 12 differentially expressed genes (DEGs) were identified during the restriction or recovery periods, respectively (*P* < 0.05). The Gene Ontology (GO) and Kyoto Encyclopedia of Genes and Genomes (KEGG) analysis indicated that DEGs enriched in nutrient metabolism and antioxidant capacity were down-regulated, while vessel development and immunity response-related genes up-regulated. The genes involved in metabolism of tyrosine were still down-regulated in the realimentation phase. Studies in this area indicated the accelerated growth effect of early protein restriction followed by a realimentation on growth performance of lambs and explored the transcriptomics change of liver which can help to develop feeding strategies to optimize the use of feedstuffs and in providing a new perspective for the study of early nutrition and epigenetics in later life.

## Introduction

The strategies to reduce the cost of lamb production in China include the imposition of feed restrictions when cultivated forages are scarce or expensive or feed supplements are not economical viable. Searle (1979) stated that young lambs are more vulnerable to feed restriction, particularly in the period immediately after weaning^1^. Compensatory (catch-up) growth was defined as a physiological process in which the organism accelerates its growth after a period of restricted development usually because of the restriction of feed intake^2^. This phenomenon is known to occur in many species of abalone^3^, shrimp^4^, fish^5^, pigs^6^, goats^7^ and lambs^8^. Dashtizadeh (2008) reported that there appears to be a critical period in cattle and sheep from birth to three months of age when nutritional restriction will not trigger compensatory growth^7^. Extensive studies in sheep and cattle have shown that genetic factors, the age at which the restriction is imposed, the severity and duration of the restriction, the quality of the realimentation diet and the duration of re-feeding influenced the rate of compensatory growth^9,10^. Nutrient composition in the diet is one of the factors influencing an animal’s ability to recover from the effects of under nutrition, among which the most attention has been paid to protein intake.

During the last few years, a growing number of studies focusing on the developmental origin of health and disease have identified links among early nutrition, epigenetic processes and diseases in later life^11^. The ‘developmental origin of health and disease’ hypothesis^12–14^ suggests that epigenetic marks serve as memory for exposure in early life to inadequate or inappropriate nutritional factors and induce long-term changes in gene expression, potentially leading to diseases in adulthood. Epidemiology and environmental epigenetics studies verified a lower supply of protein from human milk compared with a formula that attenuated both early weight gain and later obesity^15^. The early protein hypothesis postulates that high protein intake during infancy and early childhood results in metabolic programming for later obesity as a result of adverse hormonal responses^16,17^.

In recent years, deep sequencing of transcriptomes is increasingly being utilized with promises of having higher sensitivity in identification of the differential expression of transcripts^18^. In the current study, we established an animal model with twin lambs coupled with next-generation sequencing (NGS) technology to detect the compensation effect and the mechanism of protein deficiency followed by realimentation. Knowledge of the mechanism of protein restriction and realimentation on growth performance and gene expression alterations can help in developing feeding strategies to optimize the use of feedstuffs, additionally, it could offer a new perspective for the study of early dietary factors and nutritional epidemiology in later life.

## Results

### Growth performance and organ weight of lambs

The growth performance and liver weights of the lambs are shown in Table 1. The average body weight of lambs between the LPL and NPL groups showed no significant difference at 1 and 15 days (*P* > *0.05*). The average body weight of the LPL lambs was significantly lower than that of the NPL group at 60 and 90 days (*P* < *0.05*). The ADG of LPL lambs from 15 to 60 and 15 to 90 days was significantly lower than that of the NPL lambs (*P* < *0.05*). However, no significant difference of ADG was detected between the two groups from 61 to 90 days (*P* > *0.05*). The liver weight of the NPL lambs was higher than that of the LPL group at 60 and 90 days (*P* < *0.05*). The intake of milk replacer and starter showed no difference between the two groups (*P* > *0.05*). The crude protein intake of LPL was significantly lower than that of the NPL group (*P* < *0.05*) (Table 2).

**Table 1:** Effects of protein deficiency and realimentation on growth performance of lambs.

Items	Treatments		SEM	*P*-value
	NPL	LPL		
**Body weight (kg)**				
1d	2.37	2.29	0.04	0.294
15d	6.13	6.03	0.14	0.474
60d	16.6^a^	14.3^b^	0.48	0.001
90d	26.1^a^	24.1^b^	0.60	0.006
**Average daily gain (g/d)**				
15 to 60d	239.7^a^	189.4^b^	7.51	0.003
61 to 90d	320.3	334.3	8.10	0.449
15 to 90d	267.2^a^	241.6^b^	6.65	0.013
**Liver weight (g)**				
60d	438.3^a^	392.7^b^	12.0	0.046
90d	654.9^a^	605.4^b^	10.4	0.026

The significance was calculated using Paired T-test software (Version 9.2, SAS Institute Inc., NC, USA).

In a given row, the different small letter superscripts indicate that the difference between the two values is significant (*P* < 0.05).

NPL: normal protein level; LPL: low protein level.

**Table 2:** Intake of milk replacer, starter and its major nutrients during the restriction and realimentation phases.

Items		Groups		SEM	*P*-value
		NPL	LPL		
15–60 d	Milk replacer and its major nutrients intake/d				
	Milk replacer intake (g)	190.01	180.10	3.47	0.1898
	CP intake (g)	47.65^a^	34.63^b^	2.53	0.0014
	GE intake (MJ)	3.88	3.67	0.07	0.1724
	Starter and its major nutrients intake/d				
	Starter intake (g)	332.84	305.91	17.01	0.0957
	CP intake (g)	70.16^a^	45.95^b^	5.53	0.0046
	GE intake (MJ)	5.82	5.27	0.30	0.0703
61–90 d	Total intake (g)	1135.13	1058.48	26.26	0.1116
	CP intake (g)	239.29	223.13	5.53	0.1116
	GE intake (MJ)	19.85	18.51	0.50	0.1116
15–90 d	Total intake (g)	767.76	715.00	19.81	0.0749
	CP intake (g)	166.40^a^	137.60^b^	6.48	0.0094
	GE intake (MJ)	29.55	27.45	0.73	0.0722

### Analysis of RNA deep sequencing data

In this study, 40,057,846 to 58,311,854 clean reads of 100 bp for each sample were obtained. Approximately 78% of the clean reads could be mapped to sheep chromosomes and approximately 77% of the reads in each sample were uniquely mapped to the sheep genome. The number of multiplied mapped reads was <3.18% (Table S1). The correlation of transcript expression between samples is the most important indicator for reliability of experimental results and the sampling rationality. Pearson correlation analysis showed that the FPKM values between these groups were highly correlated (r = 0.87–0.92, r < 0.01) (Fig. S1).

### Identification of differentially expressed genes

The total number of genes expressed in the liver ranged from 15,702 to 16,657, with numbers of expressed genes being similar between the two groups. Correlations between biological replicate samples showed that the expressed genes were very highly reproducible, suggesting that a major fraction of the liver transcriptome is conserved between the groups. To better survey the biological mechanism of dietary protein deficiency and realimentation on growth performance, it is important to identify the different expressed genes between the two different stages.

**Figure 1:**
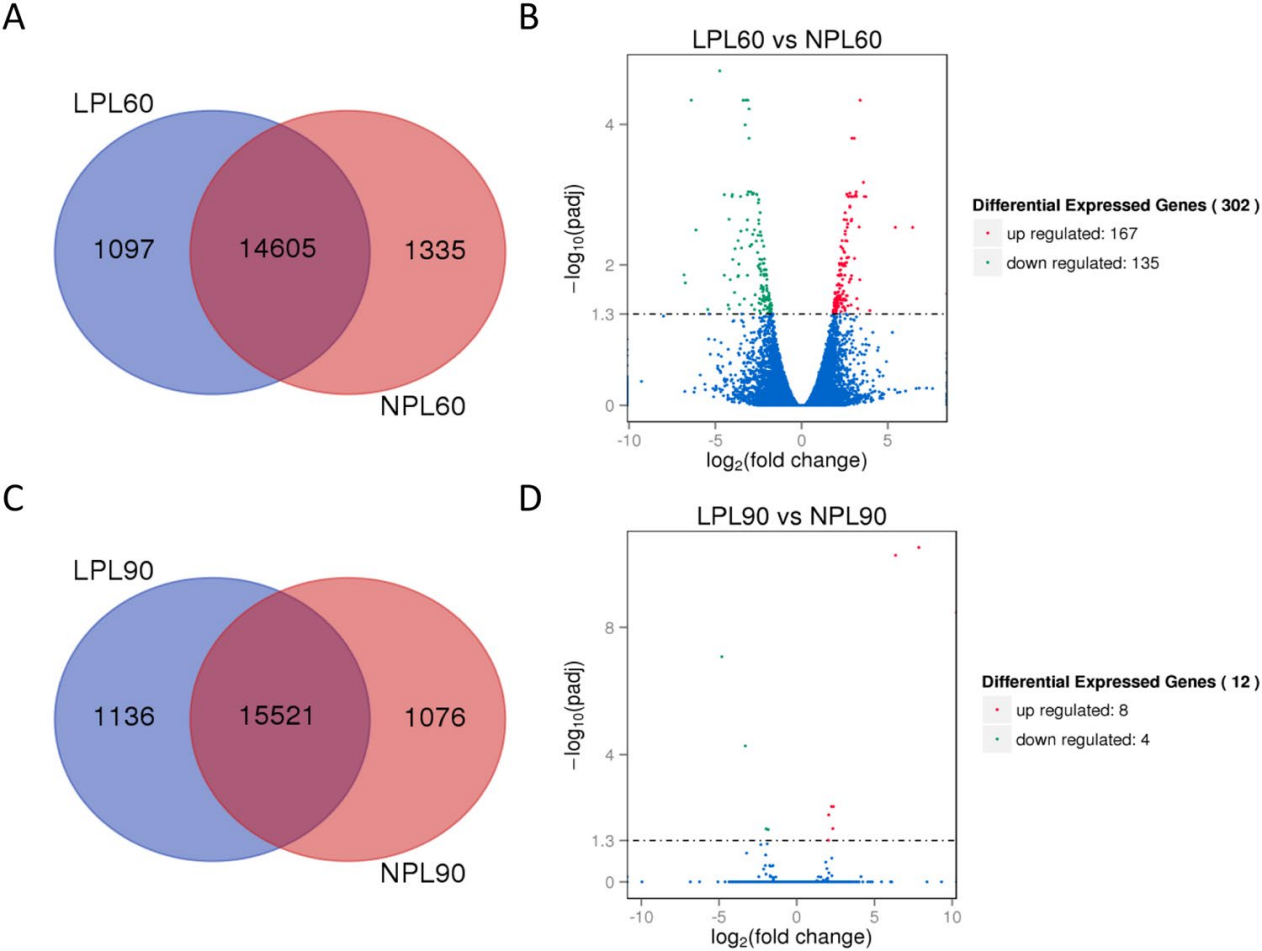
Gene expression in LPL and NPL groups at 60 and 90d. (**A**,**C**) Venn diagram showing genes only expressed in the LPL group (blue circle), only expressed in the NPL group (yellow circle), and common to both groups (intersection). (**B**,**D**) Different expressed genes between the LPL and NPL groups. The red points indicated significantly up-regulated expressed genes and the green points indicated significantly down-regulated expressed genes of LPL group. The blue points indicated that no significant difference of gene expression was found between the two groups.

The visualized profiles of DEGs (reported as the FPKM values) in this study are shown in Fig. 1. Differential gene expression in liver tissue was calculated from the clean reads using the DESeq R package. Compared to 60-old-day lambs in the NPL group (NPL60), a total of 302 DEGs were found in the liver tissue of LPL60 lambs (60-old-day lambs of LPL group) including 167 up-regulated and 135 down-regulated DEGs, accounting for 55.30% and 44.70% of the total DEGs, respectively. After realimentation of protein for 30 days, only 12 DEGs of LPL 90 group (90-old-day lambs of LPL group) were detected in the lamb livers, of which eight were up-regulated and four were down-regulated.

### Functional enrichment analysis of the DEGs

**Figure 2:**
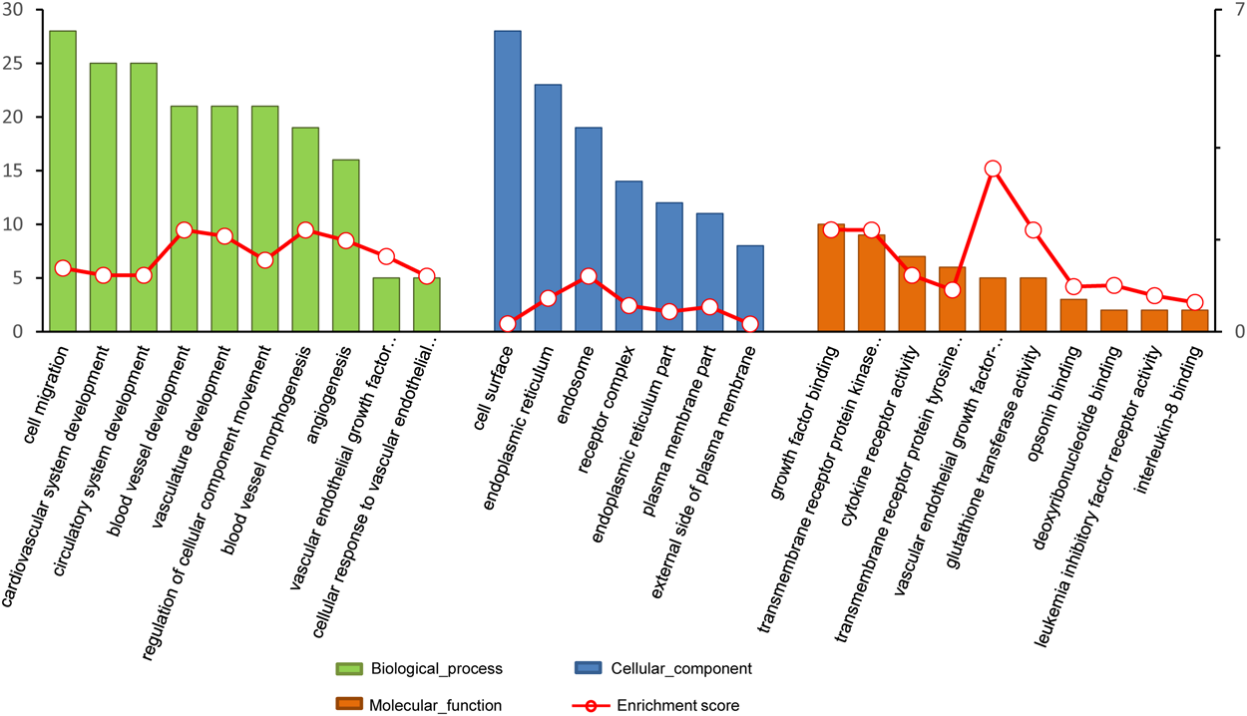
GO terms of different expressed genes for Biological Process, Cellular Components and Molecular Functions. Left ordinate represents the number of different expressed genes enriched in each term and the right ordinate represents the enrichment score (defined as 2Log10 P-value).

The DEGs were categorized according to their cellular components (CCs), molecular functions (MFs) and biological processes (BPs) (Fig. 2). The DEGs were shown to be related to biological processes such as the vascular endothelial growth factor pathway (GO: 0038084), regulation of cellular component movement (GO: 0051270), cell migration (GO: 0016477) and vasculature development (GO: 0001944) that contained several sub-terms. Molecular functions of the annotated proteins were mainly associated with four GO terms. The term vascular endothelial growth factor-activated receptor activity (GO: 0005021) is a child term of transmembrane receptor protein kinase activity (GO: 0019199), combining with vascular endothelial growth factor (VEGF) and transmitting the signal across the plasma membrane to initiate a change in cell activity. The term growth factor binding (GO: 0019838) describes interacting selectively and non-covalently with any growth factors, proteins or polypeptides that stimulate a cell or organism to grow or proliferate. The term glutathione transferase activity (GO: 0004364) involved in the catalysis of a redox reaction and regulation of the antioxidant capacity.

In addition, KEGG pathway enrichment and protein-protein interaction network (PPI) analyses were used to determine the overrepresented biological events and to provide a primary overview of the liver transcriptome that is influenced by dietary protein deficiency. Pathway enrichment analysis showed that 302 differential genes participated in 153 pathways. Among the 153 pathways that were identified, 135 down-regulated genes of LPL60 group were significantly enriched in 10 pathways that were involved in metabolism and immune competence (corrected *P*-value < 0.05; Table 3). A total of 167 up-regulated genes were mapped to 106 KEGG pathways, of which only the cytokine-cytokine receptor interaction pathway was significantly enriched (*P*-value < 0.05). After realimentation for 30 days, only the pathway of tyrosine metabolism was significantly enriched between LPL90 and NPL90.

**Table 3:** KEGG enrichment analysis of different expressed genes.

KEGG pathway	DEGs	BGs	Corrected *P* value	KEGG ID
**Significant enrichment pathway based on the down-regulated DEGs (LPL60** ***vs*** **. NPL60)**				
Chemical carcinogenesis	10	73	5.07E-09	oas05204
Metabolism of xenobiotics by cytochrome P450	9	62	1.47E-08	oas00980
Drug metabolism-cytochrome P450	7	60	3.65E-06	oas00982
Glutathione metabolism	5	53	4.61E-04	oas00480
Steroid hormone biosynthesis	5	66	9.85E-04	oas00140
Arachidonic acid metabolism	5	77	1.63E-03	oas00590
Glycine, serine, and threonine metabolism	4	42	1.72E-03	oas00260
Metabolic pathways	18	1256	5.36E-03	oas01100
Nucleotide excision repair	3	43	2.15E-02	oas03420
Ovarian steroidogenesis	3	56	3.93E-02	oas04913
**Significant enrichment pathway based on the up-regulated DEGs (LPL60** ***vs*** **. NPL60)**				
Cytokine-cytokine receptor interaction	12	235	1.14E-03	oas04060
**Significantly enriched pathway based on the down-regulated DEGs (LPL90** ***vs*** **. NPL90)**				
Tyrosine metabolism	1	34	5.65E–03	oas00350

DEGs: differentially expressed genes involved in the named KEGG pathway. BGs: all genes involved in the named KEGG pathway. DEG/BG: DEGs as a percentage of BGs.

**Figure 3:**
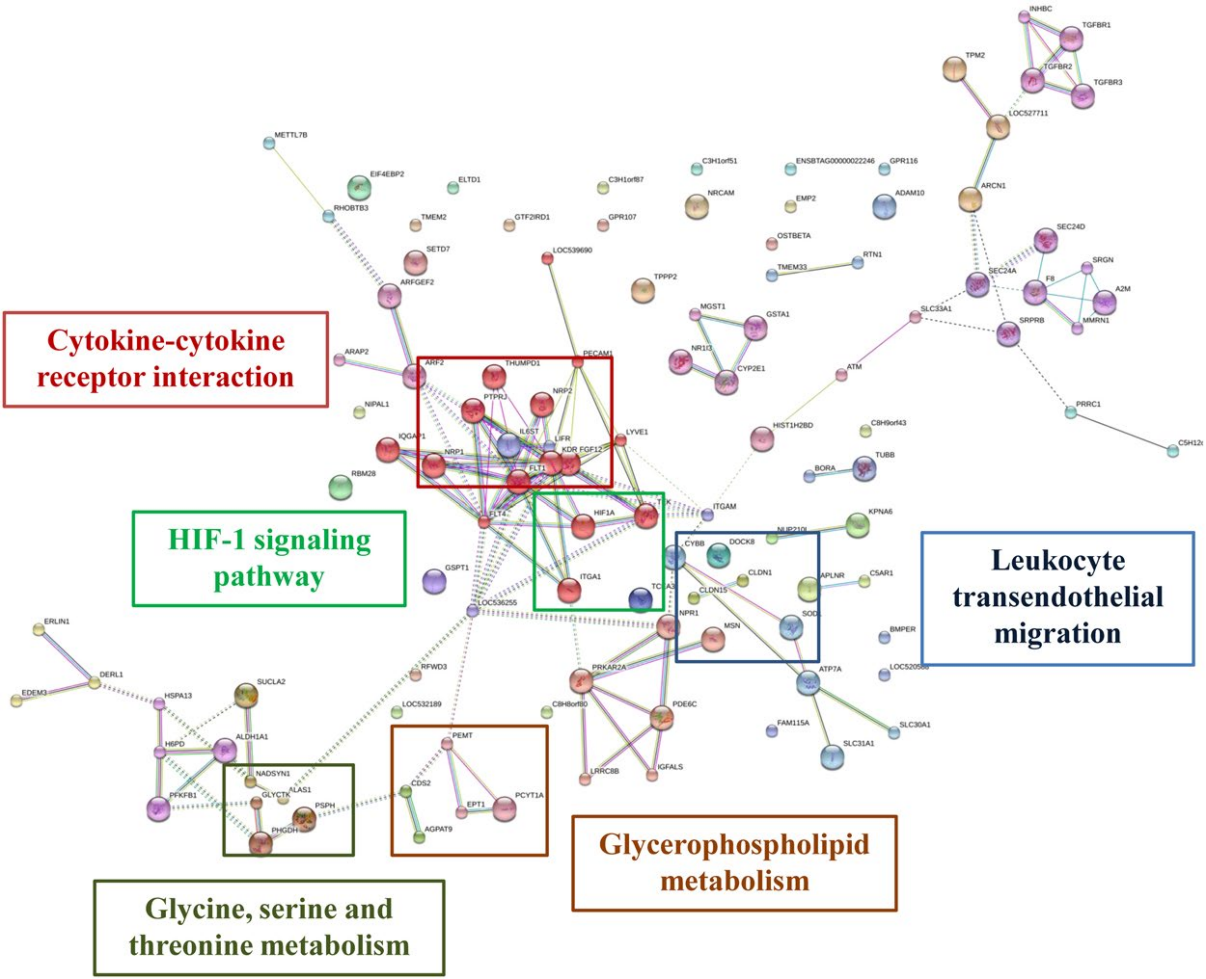
STRING analysis shows that DEGs are involved in known and predicted protein-protein interactions. STRING is used to analyse the DEGs in liver tissue between NPL60 and LPL60. Lines of different colours represent seven types of evidence used in predicting associations. Red line: fusion evidence; green line: neighbourhood evidence; blue line: co-occurrence evidence; purple line: experimental evidence; yellow line: text mining evidence; light blue line: database evidence and black line: co-expression evidence.

With DEGs as seed nodes, a protein-protein interaction network was constructed using the STRING Database version 9.0 (Fig. 3). The results showed that DEGs were mainly enriched for glycine, serine and threonine metabolism, glycerophospholipid metabolism, cytokine-cytokine receptor interaction, HIF-1 signalling pathway and leukocyte transendothelial migration.

### Real-time quantitative PCR validation of RNA-seq results

**Figure 4:**
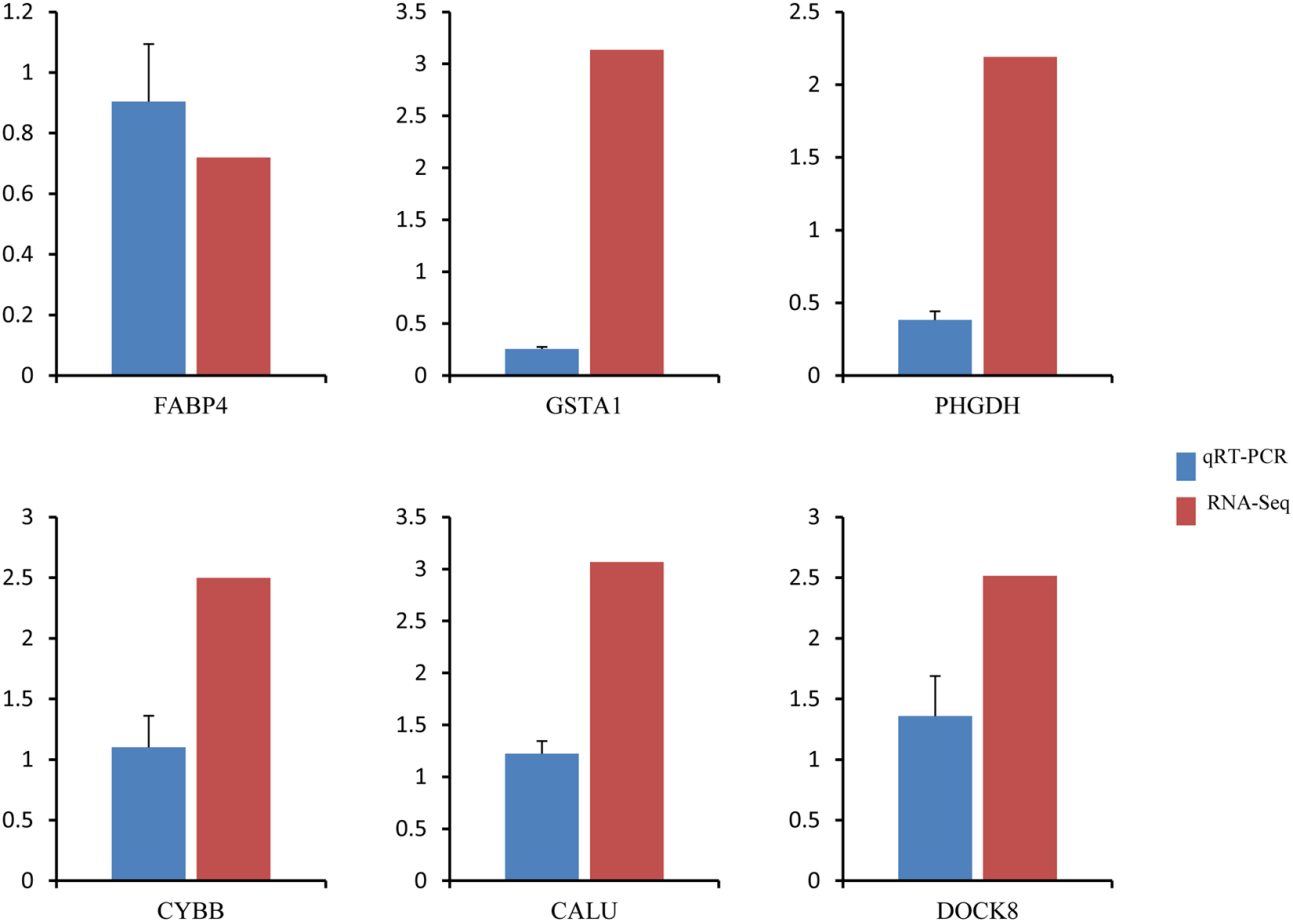
Comparison of mRNA expression ratios between LPL60 and NPL60 groups for selected genes.

We performed quantitative real time RT-PCR (qRT-PCR) assays using independently collected samples that were in the same age as those used for the RNA-seq analysis. Among the six randomly selected DEGs, three genes showed higher expression and three genes displayed lower expression in LPL60. As shown in Fig. 4, all six genes showed the same expression patterns in the qRT-PCR assays as in the RNA-Seq data, indicating that the RNA-Seq data were highly reliable.

## Discussion

Compensatory growth is manifested in the ability of animals previously restricted in feed intake to outgain their better counterparts when given free access to good quality feed. In the current study, the average daily gain and body weight of lambs decreased significantly during the protein restriction period. Compared to the NPL group, the ADG of lambs in the LPL group exhibited an accelerate effect (increased by 4.37%) after realimentation for 30 days, but there is no statistical differences between treatments. Hornick (2000) reported that compensatory growth requires an adaptation period whose duration varies from one species to another, and it takes about one month in ruminants^2^. Dashtizadeh (2008) reported that compensatory indices after realimentation of the 45-, 60- and 75-day restricted goats were 0.06, 44 and 95%, respectively^7^. The restriction period and the relimentation period were 60d or 30d, respectively. It is the adaptation period after restriction period that might attribute to the none statistical difference in the ADG of lambs. This observation is consistent with analogous trials on the compensatory growth response of goats and chickens. Higher feed intake after a period of feed restriction has been reported^19^, whereas other studies have shown that feed intake does not increase after a period of feed restriction^20,21^. The apparent contradiction of feed intake arises from the diversity of factors including breed, sex, age and quality of realimentation diet. In this study, the total intake of milk replacer and starter of NPL group showed an increased trend which indicated the similarity differences in GE intake between the two groups. It has been reported that incremental in the protein intake increases the feed intake which is due to an increase in N supply to the rumen microorganisms^22^.

As the major organ for nutrient metabolism, the liver plays an important role in the regulation of metabolic activity and physiological homeostasis of organisms. The liver weight of NPL lambs was higher than that of the LPL group during the periods of restriction and realimentation. Several studies on goat and beef heifers have reported decreases in the weights of empty stomach, intestines and liver as a result of feed restriction of young animals^23,24^. This finding is in agreement with the principle that young animals may require high protein and energy intake to maintain the organ development. This observation is in agreement with similar trials on gastrointestinal development that digestive tract tissues are affected by changes in protein intake^25,26^ and nutrient restriction^27,28^.

Next-generation sequencing (NGS) technologies have brought great advances for genomic studies that provided a large amount of data and increased the possibility of recovering important biological information from transcriptomes. In this study, RNA-seq was applied to analyze the liver transcriptome changes of lambs in response to protein deficiency and realimentation. In the present study, 78.01% of the clean reads could be mapped onto the *O. aries* reference genome, and the number of multiply mapped reads was below 3.18%. We obtained a total of 23028 Unigenes with high assembly integrity that were suitable for gene functional analysis. Analysis of Pearson correlation coefficients between two samples showed an elevated correlation of 0.92 among specimens, suggesting that transcriptome analysis results were highly reliable.

The liver is the primary organ of nutrients metabolism while the vessel is the central hub through which respiratory gases, nutrients, and wastes are exchanged. The exchange between the liver and the vessel is dependent upon blood flow, and blood flow rates are in turn dependent in large part upon vascularization of the liver. Reduction in the liver weight of lambs was related to a decrease in oxygen consumption by the liver and hepatic blood flow^29^. In this study, we have identified 302 DEGs during the protein restriction phase. GO enrichment analysis indicated that DEGs involved in biological processes were mainly implicated in vessel development and morphogenesis, and the related genes (*FLT4, KDR, BMPER,PTPRG,TGFBR2 et al*.) were up-regulated under the protein restriction condition. The protein-protein interaction network analysis also verified that DEGs enriched in the HIF-1 signalling pathway were related to the regulation of oxygen homeostasis and angiogenesis. These observations are consistent with analogous trials that a 30% dietary restriction (DR) enhanced vessel maturation in mouse CT-2A astrocytoma^30^. Redmer (2012) also reported that the mRNA expression of five angiogenic genes was up-regulated in the foetal cotyledon of pregnancies that have a low intake at 90 d of gestation, commensurate with blood vessel remodeling^31^.

Previous studies indicated that a moderate feed restriction resulted in appreciable changes in the metabolism of the liver and gastrointestinal tissues^32^. Similar results have been reported by several other authors. Doreau (2003) reported that the decrease in feed and nutrient intake with a long-term protein deficit might be attributed to a complex set of metabolic alterations related to ruminal digestion and fermentative activities^33^. KEGG analysis of this study showed that the pathways involved in the metabolism of lipids, amino acids and xenobiotics were down-regulated during the protein restriction phase. After realimentation for 30 days, the metabolism of tyrosine does not totally resume to the contrasting group’s normal level yet. It should be noted that metabolic processes are most likely affected by nutrient s intake.

Oxidative stress occurs when cellular ROS overwhelms the endogenous antioxidant defense capacity and thus, redox homeostasis is not maintained and is suggested to influence the development of metabolic syndrome and neurodegenerative disorders^34,35^. Previous studies reported that dietary protein and energy deficiency in young and mature mice decreases antioxidant capacity, as is represented in decreased superoxide dismutase (SOD), glutathione peroxidase (GSH-Px), and catalase (CAT) activities^36,37^. Transcriptome data of this study showed that expression of genes related to oxidoreductase activity, glutathione transferase activity, and oxidation-reduction process were significantly down-regulated, indicating that protein restriction significantly decreased the antioxidant capacity of lambs. This is in agreement with previous studies. He (2012) demonstrated that a decreased plasma antioxidant capacity in 28-old weaning goats caused by 4 wks of protein or energy restriction could be completely reversed after 9 wk of removing nutrient restriction^38^. In this research, the expression of antioxidant related genes returned to normal levels after 30 days of realimentation.

It is well known that cytokines are crucial intercellular regulators and mobilizers of cells that are engaged in innate as well as adaptive inflammatory host defenses, cell growth, differentiation, cell death, angiogenesis, and development and repair processes aimed at the restoration of homeostasis^39^. KEGG and PPI analyses verified that the genes enriched in cytokine-cytokine receptor interactions were up regulated during the protein restriction phase, indicating that protein deficiency affected the immune status. Deng (2004) also reported that the immune system develops rapidly in early life but is easily affected by malnutrition in the prenatal period, with long-term consequences^40^. After the recovery period, the expression of genes involved in cytokines was similarity with the normal group. However, a total of 12 genes showed a significant difference from the normal group after the relimentation for 30 days, especially the methyltransferase gene that plays an important role in DNA methylation. The ‘developmental origin of health and disease’ hypothesis indicated that the epigenetic marks serve as a memory for exposure in early life to inappropriate nutritional factors, inducing long-term changes in gene expression, potentially leading to diseases in adulthood. Thus, whether early protein deficiency and realimentation invoked the modification of epigenetics should be researched in the future.

In conclusion, the results of the present study indicated the accelerated growth effect of early protein restriction followed by a realimentation and explored the transcriptomics change of liver coupled with RNA-sequencing technology for the first time. The adaptation period after restriction period that might attribute to the none statistical difference in the ADG of lambs. The results indicated that early protein restriction decreased the nutrient metabolism and antioxidant capacity, increased the vessel development and the immunity response and verified the related expression of genes. However, follow-up studies are necessary to further clarify the correlation of early protein intervention and epigenetic regulation.

## Materials and Methods

### Ethics statement

This research was conducted at Jiangyan Hailun Animal Husbandry Co., LTD, in Jiangsu, China. All experiments were carried out according to the Regulations for the Administration of Affairs Concerning Experimental Animals published by the Ministry of Science and Technology, China, in 2004. All experiments were approved by the Chinese Academy of Agricultural Sciences Animal Ethics Committee, and humane animal care and handling procedures were followed throughout the experiment.

### Animals and diets

Sixteen pairs of twins of neonatal ram Hu lambs, born on the same day, with an average body weight of 2.3 ± 0.20 kg were randomly divided into two groups after weaning off ewe’s milk on the 15th day (6.1 ± 0.56 kg). Sixteen lambs (one lamb from each pair of twins) separated from the ewes were fed with milk replacer and starter with a normal protein level (NPL; protein level in the milk replacer and starter, 25 and 21%, respectively), and the other group was fed a diet with a low protein level (LPL) in the milk replacer (19%) and starter (15%). Both diets provided an equivalent amount of energy. The components and chemical composition of the basal diet are shown in Table 4. Each group was fed the assigned milk replacer and starter from 15 to 60 days, after which the lambs in both groups were provided with only starter containing 21% protein until day 90. The amount of milk replacer fed to each lamb was 2% (w/w) of its weight, and the appropriate starter was given *ad libitum* to each group. The intake of milk replacer and starter were measured individually.

**Table 4:** Ingredients and nutrient composition of the milk replacer and starter (dry matter basis).

Items	Diet			
	Starter		Milk replacer^b^	
	NPL	LPL	NPL	LPL
**Ingredients (%)**				
Corn	49.10	65.90	—	—
Soybean	28.90	12.10	—	—
Wheat Bran	8.00	8.00	—	—
Alfalfa	10.00	10.00	—	—
Premix^a^	4.00	4.00	—	—
Total	100.00	100.00	—	—
**Nutrient level**				
DM	89.65	90.36	97.73	97.94
CP	21.08	15.02	25.08	19.23
DE (MJ/kg DM)	13.06	13.06	18.38	18.32
EE	1.70	1.70	11.18	12.98
Ash	7.40	6.50	5.29	4.85
NDF	15.46	14.79	—	—
ADF	7.84	7.47	—	—
Ca	0.96	0.98	1.13	1.09
P	0.57	0.51	0.51	0.48

^a^The premix provides the following per kg of diet: Fe, 22.1 g; Mn, 9.82 g; Cu, 2.25 g; Zn, 27.0 g; Se, 0.19 g; I, 0.54 g; Co, 0.09 g; vitamin A, 3.20 g; vitamin D3, 0.80 g; vitamin E, 0.4 g.

^b^The milk replacer used in this study was a patented product (application number: 02128844.5). The basic component of milk replacer include soy milk powder, milk power, whey power, gelatinized starch, CaCO_3_, CaHPO_3_, NaCl, lysine, methionine, tryptophan, threonine and other mineral and vitamin premix.

NPL, normal protein level; LPL, low protein level; DM; dry matter; CP, crude protein; DE, digestible energy; EE, ether extract; NDF, neutral detergent fiber; ADF, acid detergent fiber; Ca, calcium; P, phosphorus. –,not present or can be ignored.

Bodyweight was measured before morning feeding on days 1, 15, 60, and 90. Four pairs of twins of each group were slaughtered on days 60 and 90, separately. The samples of the liver tissue were quickly harvested and frozen in liquid nitrogen for total RNA extraction.

### Isolation of total RNA and quality assessment

Total RNA was extracted from each sample using TRIzol reagent (Invitrogen, CA, USA), and DNA was removed by digestion with RNase-free DNase (New England Biolabs, MA, USA) for 30 min at 37 °C. RNA degradation and contamination was monitored on 1% agarose gels. RNA purity was checked using the NanoPhotometer® spectrophotometer (IMPLEN, CA, USA). RNA concentration was measured using the Qubit® RNA Assay Kit in a Qubit® 2.0 Fluorometer (Life Technologies, CA, USA). RNA integrity was assessed using the RNA Nano 6000 Assay Kit of the Agilent Bioanalyzer 2100 system (Agilent Technologies, CA, USA).

### mRNA library construction and sequencing

Transcript library construction, clustering, and sequencing were performed at the Novogene Bioinformatics Institute, Beijing. A total amount of 3 μg of RNA per sample was used as the input material for the RNA sample preparations. The mRNA was purified from total RNA using poly-T oligo–linked magnetic beads (Invitrogen, USA). Fragmentation was carried out using divalent cations at an elevated temperature in NEB First Strand Synthesis reaction buffer (5×) (NEB, USA). RNA fragments were used to synthesize the first and second strand cDNAs. The libraries were generated by PCR after selection and purification of the cDNA fragments. The amplified DNA was purified and the quality of each library was assessed on the Agilent Bioanalyzer 2100 system. Finally, the cDNA libraries were sequenced on the Illumina Hiseq. 2000 platform and 100 bp paired-end reads were generated. Raw data presented in this paper were submitted to the NCBI Short Read Archive: the accession number was *SRX2160967*.

### Read quality control and sequence mapping to the reference genome

Raw sequence data were transformed by base calling into sequence data and were stored in the fastq format. Quality control and read statistics were determined using FASTQC, and the quality scores Q20 and Q30 and the GC content of the clean data were calculated. All RNA-Seq reads were mapped on the reference sheep genome (Oar_v4.0) using TopHat v2.0.1 software^41^ with default parameters after removal of sequencing adaptation and low-complexity reads. Gene expression was estimated by calculating the expected number of Fragments Per Kilobase of transcript sequence per millions of base pairs sequenced (FPKM)^42^. Positive gene expression was defined by RPKM values greater than 1, while values less than 1 were excluded.

### Functional enrichment analysis of differentially expressed genes

The differential expression analysis of two groups was performed using the DESeq R package (1.18.0)^43^. The resulting *P*-values were adjusted using the approach of Benjamini and Hochberg for controlling the false-discovery rate. Genes with |log2(fold-change)| > 1 and an adjusted *P*-value < 0.05 found by DESeq were classified as being differentially expressed^44^. The GO enrichment analysis of the DEGs was implemented using the GOseq R package; GO terms with corrected *P*-values < 0.05 were considered to be significantly different^45^. GO-enrichment analysis was performed using Fisher’s exact test in Blast2GO software. KOBAS software was used to identify the statistical enrichment of DEGs in KEGG pathways^46^.

### Validation of RNA-seq data by quantitative real-time RT-PCR

To validate changes in transcript levels between groups, six genes which involved in in nutrient metabolism and antioxidant capacity, including *FABP4, CYBB, GSTA1, PHGDH, CALU* and *DOCK8*, were selected and quantified using qRT-PCR. Primers for qRT-PCR were designed using the Primer 5 software and were synthesized by Tsingke Biotech. Total RNA was extracted from liver tissue and was converted to cDNA using a Revert Aid First Strand cDNA Synthesis Kit (Thermo Fisher Scientific, Waltham, MA, USA) according to the manufacturer’s protocol. QRT-PCR analyses were performed on an iQ5 real-time PCR detection system (Bio-Rad; Hercules, CA). The glyceraldehyde-3-phosphate dehydrogenase (GAPDH) gene was used as an internal control to normalize the expression data^47^. Each qRT-PCR experiment was repeated three times. The relative expression of genes was calculated using the 2^−△△Ct^ method, and the standard deviation was calculated between three biological replicates^48^. The gene specific primers were listed in Supplemental Table S2.

Additionally, the data of body weight, average daily gain, and liver weight were analyzed by the independent sample t-test, all statistical analyses were performed by using SAS (version 9.1, SAS Institute, Inc., Cary, NC, USA; 2004). Treatment differences with *P* < 0.05 were considered statistically significant and 0.05 ≤ *P* < 0.10 was designated as a tendency.

## Electronic supplementary material

Supplementary material
