# Supplementary material for: Effects of dietary protein restriction followed by realimentation on growth performance and liver transcriptome alterations of lamb
Source: Sci Rep. 2018 Oct 12;8:15185. doi: 10.1038/s41598-018-33407-w (PMC6185953; doi:10.1038/s41598-018-33407-w)
Supplement: Supplementary file 1 — Supplementary material [file 41598_2018_33407_MOESM1_ESM.pdf]

Effects of dietary protein restriction followed by realimentation on growth performance and liver transcriptome alterations of lamb

K. Cui[#1], B. Wang[#1], T. Ma[1], B. W. Si[1], N. F. Zhang[1], Y. Tu[1], Q.Y. Diao[1]*

1 Feed Research Institute, Chinese Academy of Agricultural Sciences, National Engineering Research Center of Biological Feed, Beijing, China

*Corresponding author. E-mail: diaoqiyu@caas.cn

[#] These authors contributed equally to this work.

Figure S1. Pearson correlation analysis of the samples

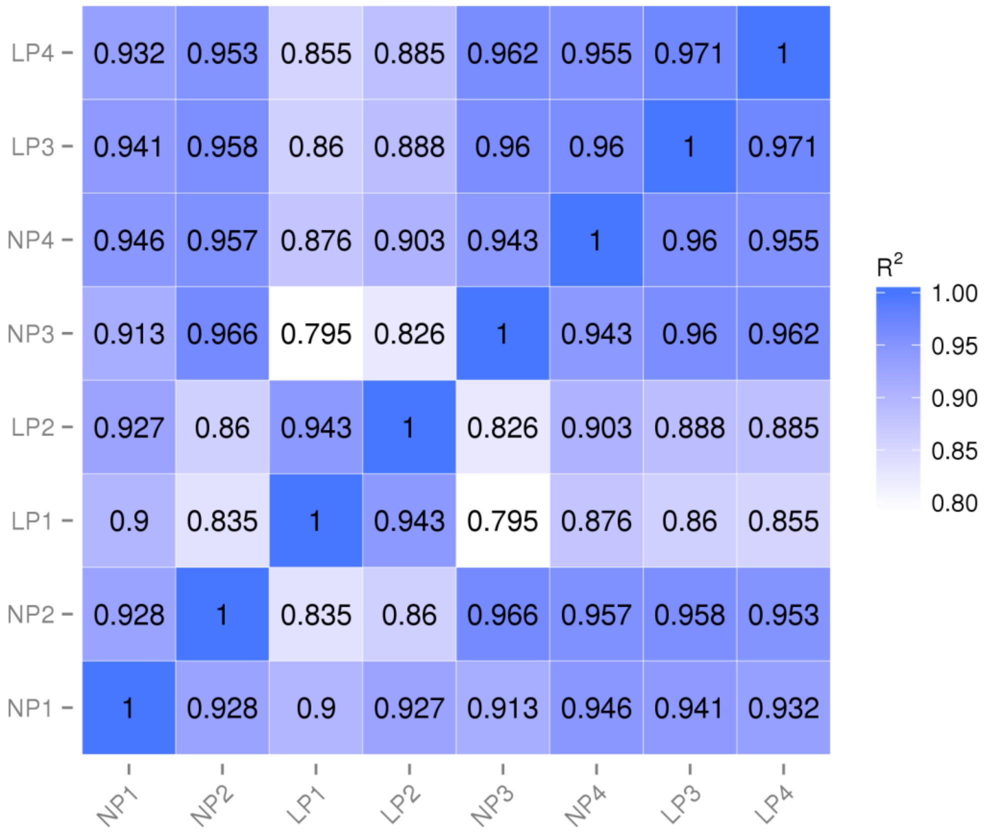

Table S1. Quality of the sequencing data

Treatment	Sample name	Raw reads	Clean reads (%)	Q_{20} (%)	Total mapped reads (%)
NPL60	NP1	59 304 426	57 538 562 (97.02)	96.40	45 118 793 (78.41)
	NP2	52 921 108	50 561 072 (95.54)	95.47	39 244 146 (77.62)
LPL60	LP1	55 080 900	53 703 926 (97.50)	96.61	41 846 795 (77.92)
	LP2	41 189 720	40 057 846 (97.25)	96.33	31 910 236 (79.66)
NPL90	NP3	62 798 110	60 592 144 (96.49)	96.13	46 750 467 (77.16)
	NP4	42 917 164	41 617 614 (96.97)	96.41	32 459 354 (77.99)
LPL90	LP3	54 828 428	53 056 652 (96.77)	96.65	41 253 420 (77.75)
	LP4	60 109 450	58 311 854 (97.01)	96.23	45 228 668 (77.56)

Q_{20}, the number of bases with a phred score >20 reported as the percentage of the total number of bases. Total mapped reads are the number of clean reads that could be mapped onto the *Ovis. Aries* reference genome.

Table S2. Sequence of primers used in quantitative real-time RT-PCR

GENE	Sequence (5′–3′)	Tm (℃)	Fragment length
PHGDH	F: ACTTTGGGGAATGCCTCCTG	60	198bp
	R: CAATCATGGTCGGCAGCATC	60	
CALU	F: GACTGGATCCTCCCCTCAGA	60	206bp
	R: ATGTGGGTTCCTCTGTCTGC	60	
CYBB	F: GACCTTGTATGGACGACCCA	60	210bp
	R: TTCCTCATGGAAGAGACCAGT	60	
DOCK8	F: CCCAGTTGCCTTGGAAAAGC	60	156bp
	R: TGGTTGTCCTGGGTGTGAAC	60	
GSTA1	F: TGCTGAAAAAGACGCCAAGC	60	217bp
	R: GGCTTTTAGGGCCTTCAGCA	60	
FABP4	F: AAGAAGTGGGTGTGGGCTTT	60	144bp
	R: CCTGGCCCAATTTGAAGGAC	60	
GAPDH	F: GTCCGTTGTGGATCTGACCT	60	245bp
	R: TGCTGTAGCCGAATTCATTG	60	
